# Insanity Can Be Cured

**Published:** 1921-09-10

**Authors:** 


*To the Editor of* The Hospital.

### "Insanity Can be Cured."

"THERE IS NO 'PROBLEM.'"

*To the Editor of* The Hospital.

Sir,—Since writing the previous articles on this topic I see that it is apparently a work of supererogation to spend much time on what does not really present any problems. Let me quote the headlines of an article from a daily paper in support of this contention. Unfortunately I shall not be allowed to use up as much space as is given to them in the original. "Lunacy *can* be cured. Simple remedy denied by obsolete laws." Let us read further : "Thousands of men and women are condemned to a living death for the rest of their existence owing to the administration of our obsolete Lunacy Laws." That "eminent mental physician" has been at it again, ladling it out hot and strong. According to him "most asylum inmates are potentially healthy-minded persons." It is the "bugs" this time. Here we have been living in ignorance that our old friends the staphylococcus, the streptococcus, old King Coli, and a gentleman called "Diphtheroid germ," have been banding themselves together, "two or more" at a time, "to lift a person over the borderline of sanity and incarcerate him in an asylum." That, as the Americans say, is "some lift." But what has our old friend, Spirochæte, done that he should be thus cold-shouldered? Evidently he, like the poor lunatic, is "left to his fate." Perhaps there isn't enough uplift about him : or the Society for the Abolition of Spirochætes is dealing with him.

Who is the Complete Ass who inspires "Our Special Representative" with such ideas : or is it that O.S.R. is looked upon as a "soft mark" on whom the *soi-disant* eminent specialist, or *spécialiste manqué*, can try his jokes? This toxic theory of the origin of certain forms of insanity is—strange as it may seem to the eminent people mentioned—by no means new. If they will consult a text-book of insanity they will probably find the toxins classified as exogenous and endogenous—and any good dictionary will give them the meanings of these terms until they are able to borrow the other volume.

It is perhaps unnecessary to waste more time over this pitiable trash : but there are one or two gems of thought to be extracted. An asylum is a "machine-made Bedlam, soulless and unproductive"—except, of course, of Cocci and Coli : it "segregates the brainsick from the world of everyday life, and that is all" : and there they are left, "in the dim half-lights of despair and unreason, to wander unguided among the strange, tortuous bypaths of delusionary dementia"—whatever that is.

Well, well, we live and try to learn. The eminent specialist does his bit : and the sagacious experts who conduct mental hospitals—not the asylums which are so designated—tell us the rest. So now there is no excuse for us if all our patients do not recover. We have only to settle the members of the Co(b)li Club.—I am, Sir, very truly yours, M. B.

